# Investigation on the Residual Stress State of Drawn Tubes by Numerical Simulation and Neutron Diffraction Analysis

**DOI:** 10.3390/ma6115118

**Published:** 2013-11-08

**Authors:** Heinz Palkowski, Sebastian Brück, Thilo Pirling, Adele Carradò

**Affiliations:** 1 Institute of Metallurgy, Clausthal University of Technology, Robert-Koch-Str. 42, Clausthal-Zellerfeld 38678, Germany; 2 Eberspächer GmbH & Co. KG, Hamburger Str. 95, Neunkirchen 66539, Germany; E-Mail: s.r.brueck@googlemail.com; 3 Institut Laue-Langevin, 6 rue Jules Horowitz, Grenoble 38000, France; E-Mail: pirling@ill.fr; 4 Institut de Physique et Chimie des Matériaux de Strasbourg, UMR 7504 UDS-CNRS, 23 rue du Loess, BP 43, Strasbourg cedex 2 67034, France; E-Mail: carrado@unistra.fr

**Keywords:** finite element method, numerical simulation, tube drawing, residual stress, neutron strain imaging

## Abstract

Cold drawing is widely applied in the industrial production of seamless tubes, employed for various mechanical applications. During pre-processing, deviations in tools and their adjustment lead to inhomogeneities in the geometry of the tubes and cause a gradient in residuals. In this paper a three dimensional finite element (3D-FE)-model is presented which was developed to calculate the change in wall thickness, eccentricity, ovality and residual macro-stress state of the tubes, produced by cold drawing. The model simulates the drawing process of tubes, drawn with and without a plug. For finite element modelling, the commercial software package Abaqus was used. To validate the model, neutron strain imaging measurements were performed on the strain imaging instrument SALSA at the Institute Laue Langevin (ILL, Grenoble, France) on a series of SF-copper tubes, drawn under controlled laboratory conditions, varying the drawing angle and the plug geometry. It can be stated that there is sufficient agreement between the finite element method (FEM)-calculation and the neutron stress determination.

## 1. Introduction

Amongst other applications, seamless tubes are used particularly where strength, resistance to corrosion, microstructure and extended product life are important design parameters. Cold drawing [1,2] is a widely applied process in industrial production, resulting in forming a high quality of the tubes under economical conditions. This process reduces dimensional inaccuracies from the pre-processing, e.g., extrusion process. However, eccentricity (E)—the deviation of the wall thickness of the tube from its average value—of the pre-tubes causes non-homogeneous deformation over the circumference during the drawing steps.

**Figure 1:**
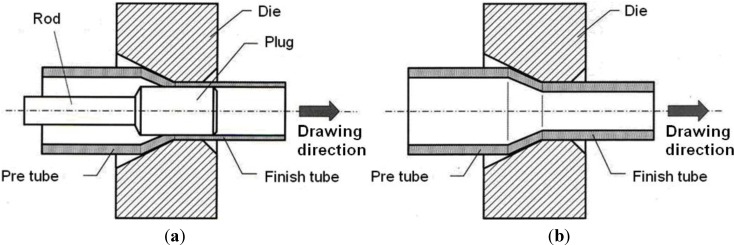
Drawing process with (**a**) a fixed plug; and (**b**) plug-less drawing.

In order to achieve the final diameter and wall thickness, the pre-extruded tubes are reduced successively in several cold drawing steps. This can be done by either drawing the tube through a die or by adding a plug, which results in better defined wall thickness and inner surface quality. The plug can be kept floating or at a fixed position by a rod. Figure 1 illustrates the process of drawing with a plug, fixed by a rod (a), and without a plug (b).

During subsequent drawing steps, the tube undergoes plastic deformation, generating residual stresses. These stresses remain after the removal of the external load [3]. Our focus is on the residual stresses due to the plastic deformation as it occurs during the cold drawing process. Eccentricity in the pre-tube influences the circumferential material flow during cold drawing and generates a complex residual stress pattern that can affect the mechanical and fatigue behaviour of the final tube [4,5,6,7].

Numerical simulation has become a very powerful technique, applied in many research fields, particularly in mechanical and civil engineering. A well-adapted model gives the chance to investigate the influences of different parameters of a process without extensive work in the laboratory or production [8]. Therefore, we developed a 3D-FEM-model that allows the linking of geometric parameters (such as wall thickness and eccentricity) with residual stress, generated by the forming process. For this development Abaqus, version 6.8 [9], was used. The quality of these models strongly depends on the input parameters and constraints. Material parameters were determined by stress-strain curves. The same importance was given to the experimental validation of the calculated results. Therefore, a set of tubes was fabricated under controlled and reproducible laboratory conditions and their stress profiles were measured by non-destructive neutron-strain-imaging. The advantage of the neutron method is that stress profiles from surfaces through the bulk material can be obtained similar to the calculation. Verification is then much more reliable than if only surface data are available as from mechanical testing or (laboratory-) X-ray measurements.

The model has been set-up to simulate the complete drawing process of plug- and plugless-drawing, starting with the introduction of the pre-tube, followed by the deformation step and stress-relaxation after leaving the tool. For a better comparison with experimental results, the output has been adapted to the location of the measuring points. However, in order to keep calculation times reasonable, simplifications had to be introduced, such as: tools (die and plug) were defined as rigid elements and the plug was kept fixed in the axial direction. Only one-half of the tube was calculated, which is justified by assuming plane symmetry. In pre-investigations, it could be stated that the position of the maximum in thickness is positioned directly opposite to the minimum.

## 2. Drawing Process and Pre-Tube Data

The investigations were done starting with raw copper tubes (DIN SF-Cu: 99.90% min. Cu, 0.015%–0.040% P), produced by extrusion to ϕ 85 mm (diameter) × 5.0 mm (wall thickness), drawn twice in a cold drawing process to ϕ 50 mm × 4.0 mm and then stress annealed before been drawn to their final dimension.

The degree of deformation in diameter (φ_d_) and wall thickness (φ_t_) are defined as: φd=ln(d1−t1d0−t0) and φt=ln(t1t0) with *d*: diameter and *t*: wall thickness. The subscripts 0 and 1 refer to the tube before and after drawing, respectively. The deformation ratio Q=φtφd is an important parameter for tube drawing describing the main direction of plastic flow [10].

The total deformation in diameter was φ_d_ = −0.53, for the wall thickness reduction φ_t_ = −0.55 and total deformation ratio *Q* = 1.04. A high *Q*-value means that the deformation results mainly out of the reduction of the wall thickness, whereas a low *Q*-value consequently indicates the main elongation of the tube coming from the reduction in tube diameter.

**Figure 2:**
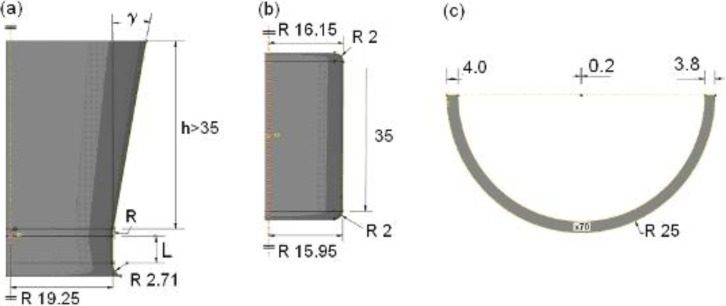
Geometrical model with example data: (**a**) die; (**b**) plug; and (**c**) thin-walled tube. All units are in mm.

In this paper two tubes, named “A” and “B”, are presented for comparison. The tubes were processed with the same die but tube A without a plug and tube B using a plug. Friction was reduced by thoroughly lubricating the tubes inside and outside with engine-oil as lubricant. The geometrical dimensions of the die, such as drawing angle, radius and length of the cylindrical part (see Figure 2), were: Drawing angle γ = 10°, radius *R* = 12 mm, cylindrical length *L* = 2 mm and inner diameter 38.50 mm. They were kept constant during the whole drawing processes.

In terms of plug-drawing, the plug was kept in a fixed position inside the die by a rod. However, it must be noted that due to gravity and the elasticity of the supporting rod, the plug can move slightly in a vertical direction. The tubes were drawn with a constant speed of 5 m/min. Tubes and plug parameters, including the geometry and deformation, are given in Table 1, where the data before drawing are compared with the ones after drawing. As before, the subscripts “0” and “1” refer to the tube before and after drawing, *d*: diameter, *t*: thickness, *A*: outer, av: average.

The plug-drawn tube B shows higher deformation—because of the increase of thickness—and greater eccentricity than tube A, drawn without a plug.

**Table 1:** Forming parameters used for drawn SF-Cu tubes.

Tube	*d*_plug_ (mm)	Deform. φ_d_ + φ_t_	*d* (mm)		Av. thickness (mm)		Δ thickness (mm)		Eccentricity *E* (%)	
			*d*_A0_ (Before)	*d*_A1_ (After)	*t*_av0_ (Before)	*t*_av1_ (After)	Before	After	Before	After
A	–	0.25	50.0	37.89	3.9	4.11	0.2	0.11	2.5	1.6
B	32.1	0.45	50.0	38.51	3.9	3.23	0.2	0.14	2.5	2.1

## 3. The 3D Elastic and Plastic FEM Model

A FEM model was developed to simulate the tube drawing process and analyse the plastic strain, as well as the residual stress state after deformation. The model was created for tubes drawn with plug and without plug, respectively.

### 3.1. Geometrical Model

Figure 2 shows the dimensions used for the model with the die (Figure 2a), plug (Figure 2b), and thin-walled tube (Figure 2c). Only one-half of the tube was modelled due to its plane symmetry and also to simplify the calculations and the analysis. In this way, the calculation time could be strongly reduced.

In the model, die and plug were regarded as being a rigid body and remained non-deformable throughout the whole simulation process. The motion and boundary condition of a rigid body were specified on a reference node and also the loads were concentrated on it.

### 3.2. Material Properties

**Figure 3:**
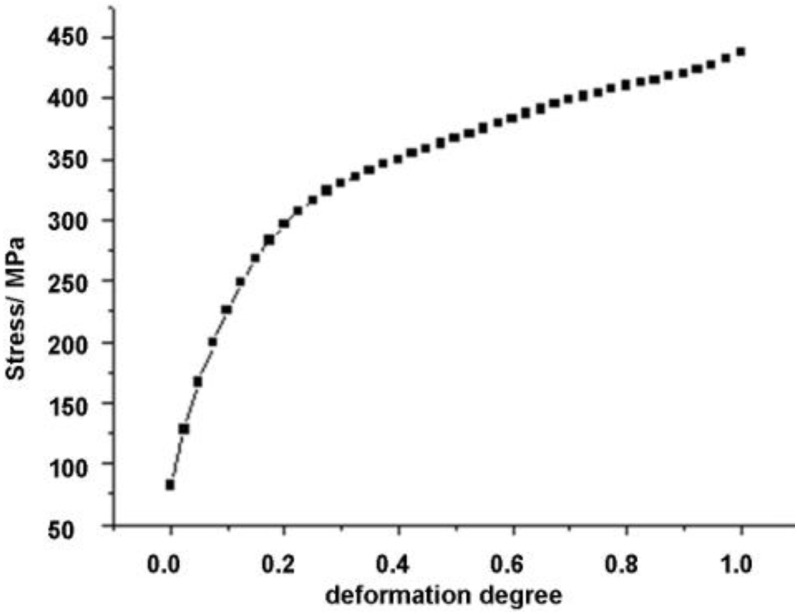
Flow curve of SF-Cu.

The material definition in the FEM model describes the behaviour of the material and has to provide all relevant data, such as density, elastic and plastic properties. The material used in the drawing experiment was copper (DIN SF-Cu). Its properties used: Young’s elastic modulus (*E* = 130 GPa), Poisson’s ratio (*v* = 0.34), density (ρ = 8960 kg/m^3^) and flow curve (flow stress *vs* degree of deformation (true strain)) by tension test, presented in Figure 3. The thermal effect of the material properties, because of dissipation, was not taken into account in this model.

### 3.3. Assembly

**Figure 4:**
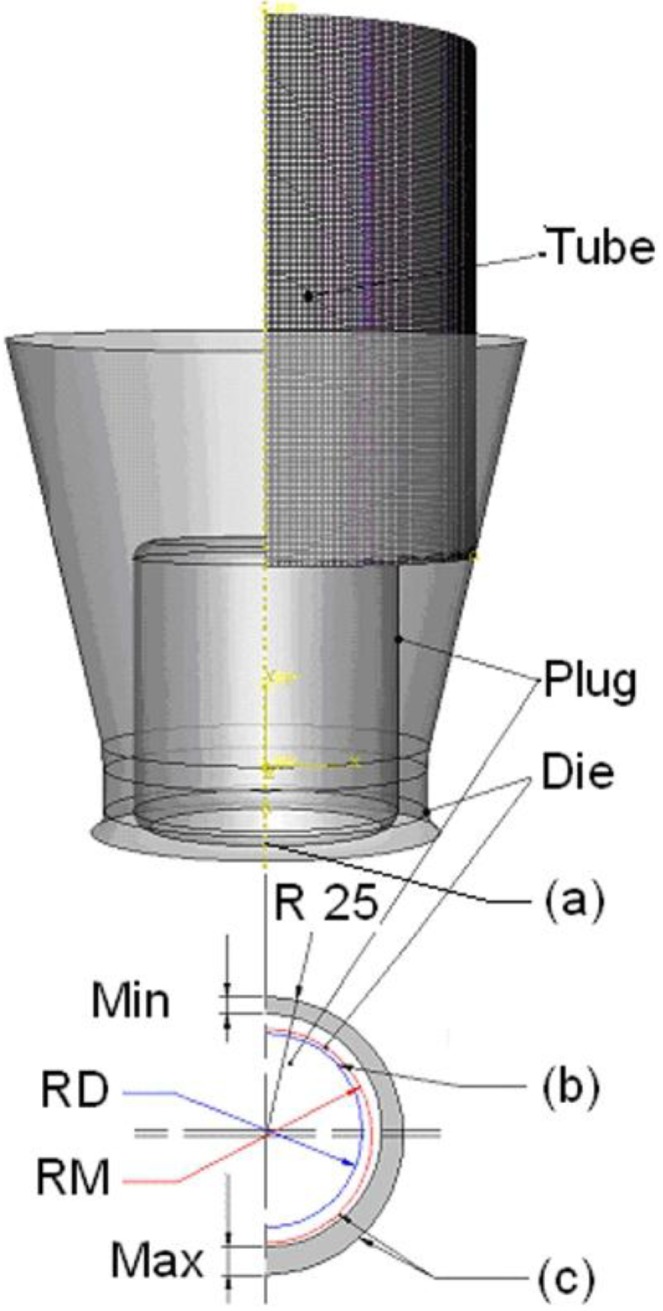
Assembly of the model: (**a**) coaxial definition, plug and inside surface of the tube; (**b**) coaxial definition, cylinder surface of the die and outside surface of the tube; and (**c**) position definition of the plug. “RD” and “RM” are the radii of the plug and the middle circle, respectively. “Min” and “Max” define the minimum and maximum wall thickness of the tube.

The assembly definition of the plug drawing model is shown in Figure 4. “RD” and “RM” are the radii of the plug and the middle circle, respectively. The arrangement of the parts tube, die and plug is described in the FEM model following the experimental drawing process.

The *first definition of assembly* is related to the coaxial conditions: in the model it was determined that the inside cylindrical surface of the die is concentric to the outer surface of the tube (Figure 4a). Moreover for the plug drawing model, the plug is assume to be coaxial with the inside surface of the tube (Figure 4b).

The *second definition of assembly* is related to the position of the plug, being special for the plug drawing model (Figure 4c). The plug position at the beginning of the calculation is close to the final position. It was not assumed, as occurs in practice that the plug keeps moving slowly in the first few seconds when starting the drawing, until it reaches the stable position. This definition can avoid some of the influence of the strong wave—that means intensive movement of the plug until it reaches a stabilized position—in the calculated process.

### 3.4. Interaction

The frictional constraints were defined in the interaction conditions. In our model, a penalty method was selected to resolve the tangential behaviour of a mechanical contact. The penalty method is a general contact type that permits some relative motion of the surfaces when they should be sticking, and it is a stiffness method for imposing frictional constraints. In this method the compressive force is proportional to the penetration of the material, using the basic concept of the Coulomb friction model. Our model combines the maximum friction (shear) stress on an interface with the contact pressure. The friction coefficient is assumed to be μ = 0.3. The frictional stress on the traction boundary is given according to a modified Coulomb law as follows:

(1) $$F_{t}=-\mu \cdot \left\|F_{n}\right\|\cdot \varphi \left(\Delta u_{t}\right)\cdot \frac{\Delta u_{t}}{\left\|\Delta u_{t}\right\|}$$

where μ is the frictional coefficient, *F_n_* is the die pressure, Δ*u*_t_ is the sliding velocity, and φ(*x*) is a modifying function describing sticking and slipping conditions as follows:

(2) $$\varphi \left(x\right)=\left\{ \begin{array}{ll} 1 & \left\|x\right\|\geq d\\ \frac{\left\|x\right\|}{d} & \left\|x\right\|\;<d \end{array}\right.$$

where *d* is an assigned value [11].

Using drawing oil as a lubricant the friction coefficient μ ranges from <0.1 to 0.3 [12]. Because of good lubrication conditions in the test and therewith a small friction coefficient, μ is taken as 0.1 for basic simulations.

A contact boundary condition is used in the model, if the distance between two surfaces is zero. There is no limited contact formulation for the amplitude of the contact pressure. If the contact pressure between the surfaces is zero or negative, the surfaces will be separated. This behaviour is characterized as being “hard” in the FEM model.

### 3.5. Boundary Conditions

**Figure 5:**
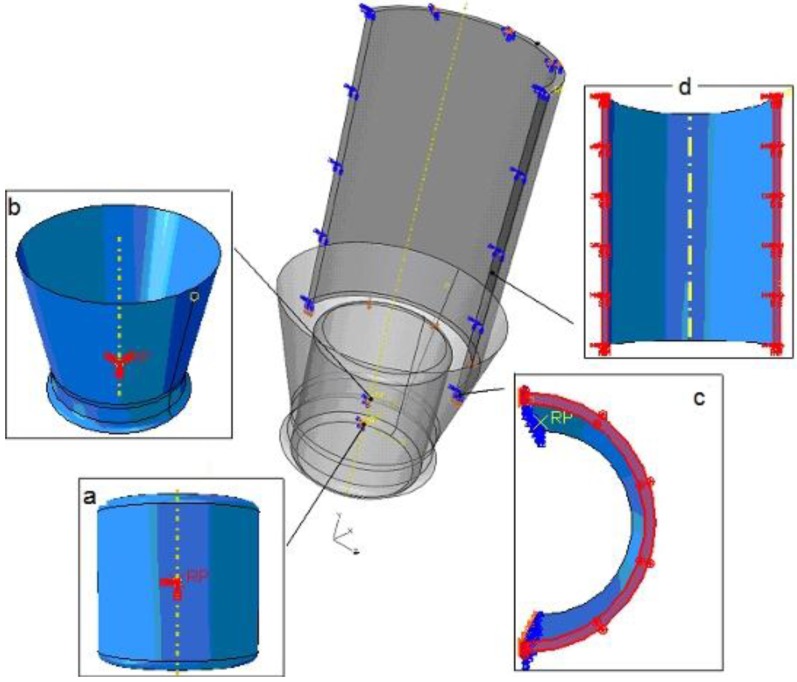
Boundary conditions: (**a**) reference point (RP) of the rigid plug; (**b**) RP of the rigid die; (**c**) tube surface; and (**d**) symmetric surface of the tube.

Boundary conditions have been used to specify the values of all basic solution variables in nodes (e.g., displacement, rotation, pressures, connector material flow, *etc.*) in the following way:

1. The plug can only move in the direction (*z*) of minimum or maximum thickness of the tube because of gravity (Figure 5 a);
2. The die is fixed in the *x*, *y*, and *z* directions during the whole drawing process (Figure 5 b);
3. The tube is set in the *x* and *z* directions until the end part enters into the die;
4. The fixation of the tube is no longer considered so that the tube can be drawn completely through the die (Figure 5 c). The drawing speed is 5 m/min, the same as for the drawing experiments. Because of the plane symmetry of the tube, it should not move in the *x* direction and also not rotate in the other directions (Figure 5 d).

### 3.6. Element and Mesh

The element type and mesh size strongly influence the calculated results. As presented by Zhang [13], hexahedral is a well adaptive element mesh for finite element analysis in the application for metal plastic forming processes. Hexahedral (C3D8R in the first order—node linear brick, reduced integration with hourglass control) is a well-adapted element for 3D contact and for forming simulations. Different mass scaling factors and element sizes were investigated to get a good compromise between the calculation time and the accuracy. For a 70 mm length and 4 mm thick tube, a global mesh size of 0.35 mm and six elements over wall thickness were chosen in the model, so the tube was generated up to 800,000 elements from FEM software.

### 3.7. Step

The step is a definition of a complete loading cycle of a simulation. Our model divided the drawing process into four steps and each step was defined as a period of time:

1. the tube being slowly brought into contact with the die;
2. the contact of the plug with the tube;
3. holding the end of the tube, so that the tube could run exactly in the range of the die and;
4. fixing of the tube end is no longer considered so that it can be completely drawn through the die.

## 4. Measurement Conditions of Strain and Residual Stress

The residual stress measurement by diffraction techniques is based on the determination of the lattice spacing, *d*, of a given crystallographic (*hkl*) by means of Bragg’s Equation. By comparing the peak position for different spatial directions with that of the unstressed sample, the strain tensor can be calculated. The stresses can be then readily determined by applying the generalized Hooke's law. This analytical procedure has been explained in detail elsewhere [14].

The residual strain measurements were performed on the strain imager for engineering applications SALSA at the Institute Laue Langevin in Grenoble, France. The instrument was chosen because it is equipped with two radial focusing collimators, which provide a very low surface error and are therefore well suited for measurements in thin walled samples.

SF-Cu has a face-centred-cubic structure with space group Fm3m and lattice parameter *a*_0_ = 0.36148 nm. The family of planes {311} was used for strain determination at a wavelength of λ = 0.165 nm. This brings the diffraction angle to 2θ ~ 98°, providing a practically equal shaped gauge volume in all measuring geometries for the axial, radial and hoop-direction. The neutron beam was defined by three radial focussing collimators, providing horizontally a beam width of 0.6 mm (FWHM) for the primary and secondary beam, and a width of 2 mm (FWHM) in the vertical direction.

**Figure 6:**
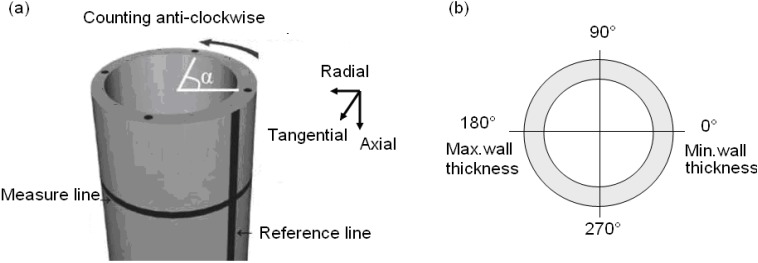
(**a**) Points of measurement and (**b**) measuring points over circumference.

Measurements of residual stress were performed in radial, hoop and axial directions (Figure 6a), a minimum of 120 mm away from tube ends to avoid measurements in the area of relaxation. Complete through thickness scans were performed at four locations on the circumference, separated by 90°. The thinnest wall thickness *t*_min_ lies at position 0° and the maximal *t*_max_ at position 180° (Figure 6b). The distance between measuring points was 0.2 mm. The precise location of the surface was determined by calculating the centroid position of the sampled gauge volume from the intensity profile [15]. The co-ordinates of the measuring points were then calculated with respect to the so found surface position. Stress values were determined from interpolated strain values at equidistant points of 0.2 mm distance.

## 5. Results and Discussion

### 5.1. Simulation Results

**Figure 7:**
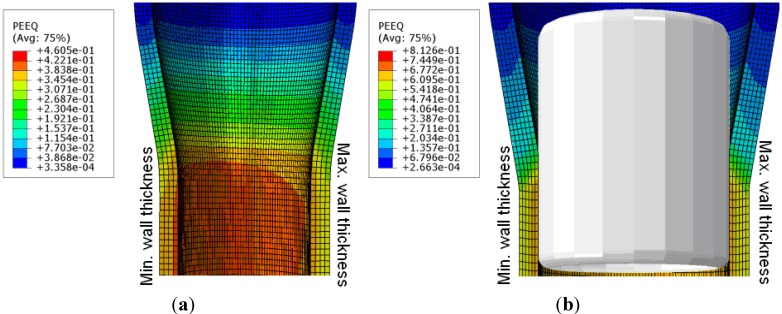
3D-FEM model for the tubes with contour plot of equivalent plastic strain (PEEQ). (**a**) Without plug and (**b**) with plug.

Figure 7 shows the calculated contour plot of the “with and without plug” drawn tubes with the geometrical data, as listed in Table 2. The contour for the tube elements represent the equivalent plastic strain (PEEQ) (without plug, blue: min = 0, red: max = 0.46; with plug, blue: min = 0, red: max = 0.81). The results show some differences between the minimum and the maximum wall thickness due to the different material flow. This influences the residual stress state in these locations.

**Figure 8:**
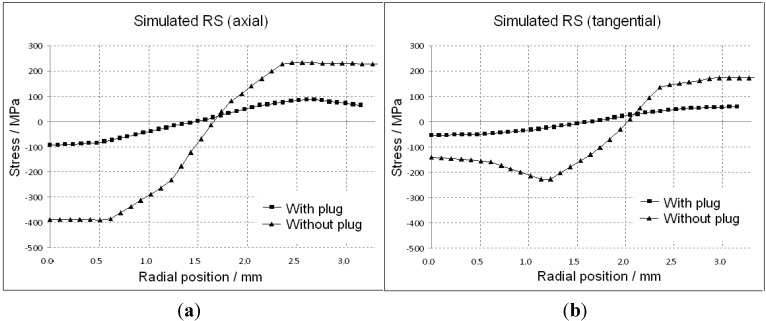
Simulated residual stresses (RS) for drawn tubes (with and without a plug), (**a**) in axial and (**b**) in tangential direction.

**Figure 9:**
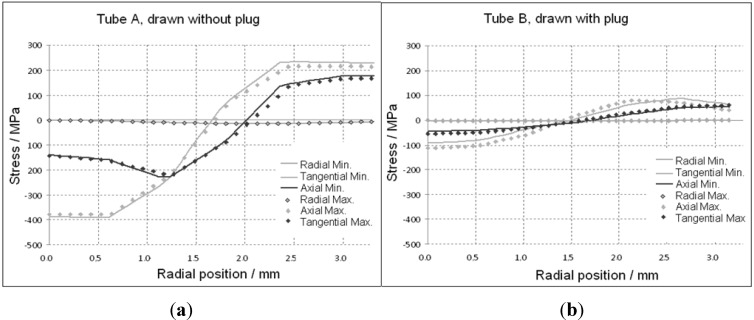
Simulated residual stresses in the position of minimum and maximum wall thickness for (**a**) drawn without and (**b**) with a plug. Radial position “0” is the inner wall surface.

The calculated residual stress profiles of the components of the principal axes of the tubes drawn with and without plug are given in Figure 8 and Figure 9. The hoop and axial directions show a gradient with tensile residual stresses at the outer surface and compressive states at the inside of the tube.

Figure 8 shows the calculated stress profiles over the wall thickness (“0”: inner surface) in axial (a), and tangential (b), directions for tubes drawn with and without a plug. The results are in agreement with other publications [16,17,18,19,20,21] stating that the stress amplitudes within the tubes, drawn without a plug, can be two to three times higher compared to the tubes drawn with a plug. Furthermore, the relationship of residual stresses between the thinnest wall thickness and maximum wall thickness for tubes drawn with and without a plug is presented in Figure 9. The full lines represent the stresses at the position of the minimum thickness and the dotted lines give the values at the maximum position. The numerical calculation shows a negligible difference between the two positions (pre-tube with 2.5% eccentricity).

### 5.2. Comparison and Evaluation of Simulation with Experiment Results

**Figure 10:**
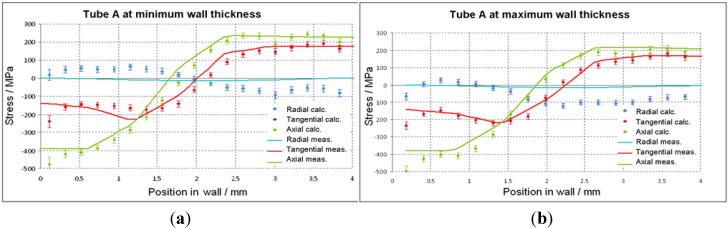
Distribution of residual stresses in tube A (without plug) at (**a**) maximum and (**b**) minimum wall thickness; dots: measurement, lines: FEM calculation.

A comparison between the experimentally measured data and the simulation curves is shown in Figure 10, illustrating the residual stress over the wall thickness state from the inside to the outside of the tubes, drawn with and without a plug at the position of maximal wall thickness. Simulation data (full line) were calculated at the same position as the experimental data from SALSA strain scanning. Furthermore, they were integrated over a volume that is comparable to the real gauge volume of the measurements. The stress level at the inner side of the tube is compressive and changes to tension at the outside. The drawn tube without plug represents a high value of stress in the inner side, and the axial component in all cases shows the highest level of residual stresses.

Experiment and simulation of axial and tangential stresses agree very well, even quantitatively. Figure 10 shows the stress profiles for the thin and thick wall. The measurements are suitable to detect even the small difference of stress levels at the outer surface, as predicted by the calculation. Only for the radial component, do the results differ between simulation and measurement clearly with max. 100 MPa in compression and 30 MPa in the tensile stress state. Nevertheless, a probable change in texture has not been taken into account up to now in the model. This might be a reason for these residual stress levels as well as the non-consideration of a tilted stress tensor in the simulation.

The difference between simulation and experimental data for tube A is listed in Table 2.

**Table 2:** Deviation between simulation and experiment evaluation of tube A.

Tube A (without plug)	Simulation	Experiment	Disagreement abs.	Disagreement in %
Average thickness (mm)	4.17	4.11	0.06	1.5
Thickness difference (mm)	0.16	0.13	0.03	23.1
Eccentricity (%)	1.9	1.6	0.3	18.8
Outer diameter (mm)	37.85	37.89	−0.04	0.1
True strain in diameter φ_d_	−0.313	−0.312	−0.001	0.3
True strain in wall thickness φ_t_	0.067	0.052	0.015	28.8
Deformation ratio *Q*	0.21	0.17	0.04	23.5

In the theoretical case of tube B, the plug and die were defined in a stable situation. The level of the simulated residual stresses in all directions was much smaller than for tube A. After comparing with the feedback results of the experiment, it was not exactly similar. From the side of using the same processing data of pre-tube and tools, it can be said that the stability of the plug is an important factor in avoiding the residual stress state of drawn tubes. The difference between the experimental and simulation data with and without the plug drawn tubes points to a question, which has to be answered in the future: what is the effect of the plug on the residual stress state in the drawing process, or what is the effect of the stability of the plug on the residual stress state in the tube drawing process?

## 6. Conclusions

Under the definitions in symmetric, material, load and boundary conditions, the FEM model simulated the drawing process of tubes with and without a plug. The variation of wall thickness and eccentricity, deformation and residual stress state were calculated. Experimental data were taken to compare with the results obtained by simulation. Some conclusions can be made:

1. The model is suitable for a tube drawing process with and without using a plug to calculate the wall thickness, eccentricity, deformation, stress and residual stress state;
2. The model delivers a good basis for the calculation of wall thickness and eccentricity for cold drawn tubes.

The results of the model for the drawing process without a plug fit well with the experimental data, nevertheless there are still discrepancies for radial stresses which need to be clarified.
